# Supplementary material for: Global Proteome Profiling of the Temporal Cortex of Female Rats Exposed to Chronic Stress and the Western Diet
Source: Nutrients. 2022 May 5;14(9):1934. doi: 10.3390/nu14091934 (PMC9103025; doi:10.3390/nu14091934)
Supplement: Supplementary file 1 [file nutrients-14-01934-s001.zip › Figure S 2.pdf]

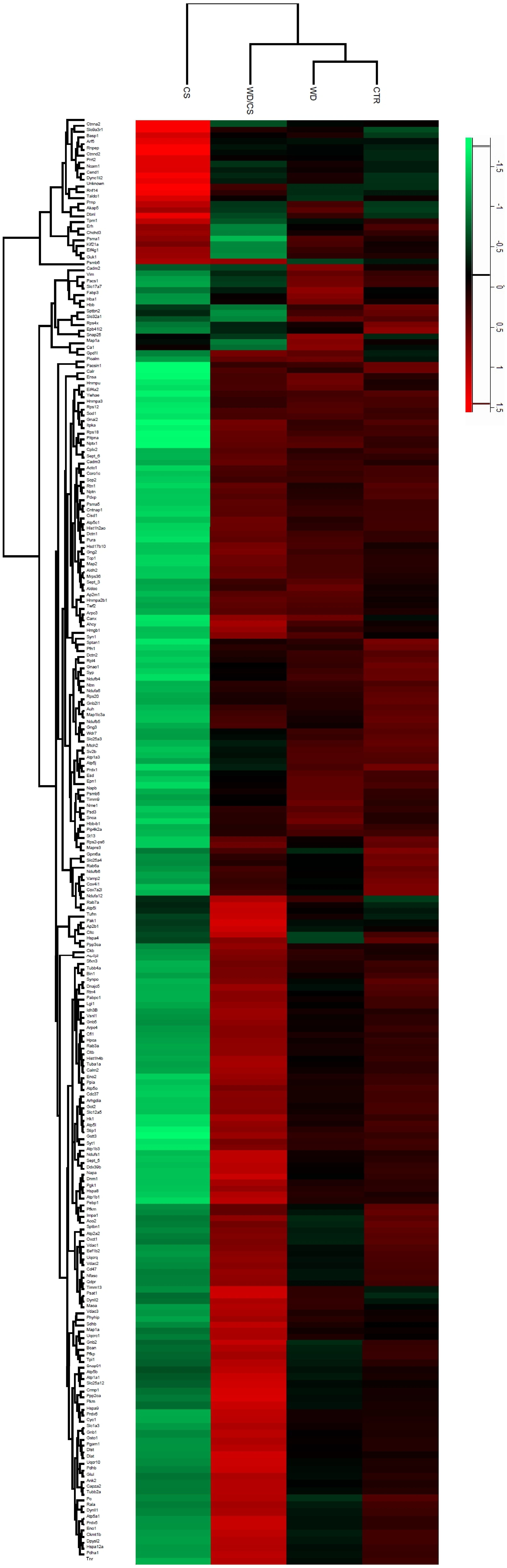

Figure S2. A heat map of the cerebrocortical proteome analyzed by the two-way ANOVA, in which green indicates low protein abundance and red indicates high protein abundance. Each column represents an average of each experimental group: CTR (n=12); control rats fed with standard diet, WD (n = 12); rats fed with western diet, CS (n=6) rats fed with standard diet and subjected to chronic stress; CS/WD (n=6) rats fed with western diet and subjected to chronic stress. The number in the color scale indicates z-score.
