# Supplementary material for: Global Proteome Profiling of the Temporal Cortex of Female Rats Exposed to Chronic Stress and the Western Diet
Source: Nutrients. 2022 May 5;14(9):1934. doi: 10.3390/nu14091934 (PMC9103025; doi:10.3390/nu14091934)
Supplement: Supplementary file 1 [file nutrients-14-01934-s001.zip › Table S 2 Table summarized fold changes.pdf]

Table S2. Table summarized fold changes and p-values from post hoc analysis of identified proteins.

	Gene name	ANOVA p-value	FC (CTR vs. WD) p-value		FC (CTR vs. CS) p-value		FC (CTR vs. WD/CS) p-value		FC (CS vs. WD/CS p-value	
F1LQ63	Tnr	0.000375	0.9181	ns	0.7561	ns	1.1508	ns	1.5219	0.0209
Q1WIM2	Cadm2	0.002757	0.9386	ns	1.3187	ns	1.2898	ns	0.9781	ns
P28073	Psmb6	0.011788	1.0286	ns	1.3603	ns	0.6422	0.0025	0.4721	<0.0001
Q91XN7	Tpm1	0.000839	1.2273	ns	1.7702	0.0002	0.9562	ns	0.5402	0.003
Q9R1Z0	Vdac3	0.021143	0.8311	ns	0.6126	ns	1.2229	ns	1.9962	ns
P97829	Cd47	0.015376	0.7813	ns	0.6526	0.0071	1.0735	ns	1.6450	0.0046
P52873	Pc	0.007681	0.9440	ns	0.8195	ns	1.1982	ns	1.4621	0.0417
Q66WT9	Picalm	0.008600	1.3751	0.0122	1.0521	ns	0.8076	ns	0.7675	ns
A0A0G2J9	Hbb-b1	0.008217	1.4053	0.0066	0.3482	<0.0001	0.8258	ns	2.3718	<0.0001
Q6AYH5	Dctn2	0.001044	1.0884	ns	0.7926	ns	1.1618	ns	1.4658	0.04
F7FKI5	Pdha1	0.001053	0.8935	ns	0.7157	0.0488	1.2334	ns	1.7234	0.0016
A0A0G2JY3	Ctna1	0.004372	1.1900	ns	1.9984	<0.0001	1.0523	ns	0.52659	0.0002
Q6IMY8	Hnrnpu	0.000006	0.9222	ns	0.6466	0.0057	0.9646	ns	1.4918	0.298
Q63363	Sptan1	0.000030	1.0477	ns	0.8172	ns	1.1376	ns	1.3921	0.089
F1LRK0	Snap91	0.013624	1.0222	ns	0.8979	ns	1.1463	ns	1.2767	ns
A0A0G2K18	Basp1	0.011191	1.4969	0.0009	3.4992	<0.0001	1.3314	ns	0.3805	0.0001
B0BN52	Mtch2	0.009762	0.8472	ns	0.5642	0.0001	0.9632	ns	1.7072	0.002
G3V7U2	Map1a	0.011724	1.0667	ns	0.9223	ns	1.1870	ns	1.2870	ns
Q5FVG4	Slc25a22	0.004211	0.7990	ns	0.6248	0.0025	0.7392	ns	1.1831	ns
D3ZZK1	Rps20	0.000185	0.9373	ns	0.5364	<0.0001	0.8262	ns	1.5403	0.0168
D3ZCR3	Hmgb1	0.013407	1.1470	ns	0.5418	<0.0001	1.1976	ns	2.2102	<0.0001
F1M9N9	Ank2	0.002239	0.9603	ns	0.8415	ns	1.2077	ns	1.4352	ns
Q1WIM3	Cadm3	0.000019	1.0413	ns	0.7256	ns	1.0469	ns	1.4428	ns
B2RYX1	Uqcr10	0.003129	0.9506	ns	0.7521	ns	1.2650	ns	1.6819	0.0028
Q6XD99	Sptbn1	0.000027	0.9996	ns	0.8321	ns	1.1181	ns	1.3438	ns

P09527	Rab7a	0.007276	0.8596	ns	0.7181	ns	0.9233	ns	1.2857	ns
E9PSV5	Psat1	0.014209	0.9160	ns	0.7998	ns	1.0733	ns	1.3419	ns
P49432	Pdhb	0.000845	0.9866	ns	0.8346	ns	1.2361	ns	1.4810	0.033
G3V846	Slc1a3	0.004732	0.9476	ns	0.8353	ns	1.2701	ns	1.5205	0.0213
Q9WVB1	Rab6a	0.000659	0.9116	ns	0.7355	ns	1.0117	ns	1.3754	ns
D3ZM33	Rps18	0.000482	1.0238	ns	0.5717	0.0002	1.0175	ns	1.7798	0.0008
X1WI37	Rps4x	0.008368	0.9124	ns	0.6841	0.0201	0.5115	<0.0001	0.7478	?
B5DFG5	Sept_6	0.000022	1.0480	ns	0.6138	0.0016	1.0775	ns	1.7555	0.011
O55215	Rps2-ps6	0.000542	1.1484	ns	0.6433	0.0051	1.0080	ns	1.5670	0.0122
O88339	Epn1	0.009499	0.9977	ns	0.5485	<0.0001	0.7788	ns	1.4199	0.0663
Q63692	Cdc37	0.003665	0.9482	ns	0.6922	0.0256	1.2195	ns	1.7616	0.001
P84092	Ap2m1	0.002161	1.0490	ns	0.7628	ns	1.0897	ns	1.4287	ns
B0BMW2	Hsd17b10	0.001258	0.9747	ns	0.7113	0.0435	1.0536	ns	1.4812	0.0336
B0BMY7	Twf2	0.008816	1.1515	ns	0.7349	ns	1.0611	ns	1.4439	ns
B0BNE5	Esd	0.003518	0.9969	ns	0.5992	0.0008	0.8314	ns	1.3875	ns
B0BNN3	Ca1	0.018704	1.8287	<0.0001	1.1216	ns	0.9379	ns	0.8363	ns
B0K020	Cisd1	0.002928	0.8300	ns	0.2933	ns	1.0105	ns	3.4455	<0.0001
B1H216	Hba1	0.006702	1.4158	0.0052	0.7543	ns	0.9444	ns	1.2521	ns
B1VKB4	Synpo	0.021201	1.0174	ns	0.7752	ns	1.1318	ns	1.4600	ns
B2GV06	Oxct1	0.013290	0.8631	ns	0.7504	ns	0.9938	ns	1.3243	ns
B2GV33	Maoa	0.014498	0.8225	ns	0.6471	0.0058	1.1813	ns	1.8256	0.0004
B2GV73	Arpc3	0.004683	1.1519	ns	0.6413	0.0047	1.1532	ns	1.7981	0.0006
B2GV79	Pdxp	0.003528	0.9855	ns	0.6864	0.0215	0.9900	ns	1.4423	ns
B2RYQ5	Erh	0.020835	0.9166	ns	1.2489	ns	0.8215	ns	0.6578	ns
B2RYT5	Cox7a2l	0.018672	0.8777	ns	0.7255	ns	0.9575	ns	1.3198	ns
B2RZ72	Arpc4	0.004113	0.9043	ns	0.6811	0.0183	1.1492	ns	1.6874	0.0026
B4F7C2	Tubb4a	0.021783	1.0019	ns	0.8360	ns	1.1408	ns	1.3646	ns
B5DEN5	Eef1b2	0.009698	0.8451	ns	0.7548	ns	1.0172	ns	1.3476	ns
B6DYQ5	Gsto1	0.001084	0.9876	ns	0.6817	0.0187	1.3709	0.0482	2.0110	<0.0001
B7X6I3	Cend1	0.015422	1.1487	ns	2.4235	<0.0001	1.0062	ns	0.4152	<0.0001
C7C5T2	Pfkp	0.003104	1.0273	ns	0.8748	ns	1.2010	ns	1.3728	ns

D3Z8I7	Gstt3	0.001601	0.8690	ns	0.4893	<0.0001	1.1848	ns	2.4216	<0.0001
D3ZAF6	Atp5j2	0.001921	0.8122	ns	0.8024	ns	1.2019	ns	1.4979	0.027
D3ZAP9	Gpd1l	0.009459	1.1775	ns	0.9671	ns	0.8274	ns	0.8556	ns
D3ZC55	Hspa12a	0.001738	0.9422	ns	0.8350	ns	1.1111	ns	1.3307	ns
D3ZSN6	Kif21a	0.000606	1.1391	ns	1.3473	ns	0.5525	<0.0001	0.4101	<0.0001
D3ZDH8	Sept_5	0.000004	0.9936	ns	0.6539	0.0075	1.1512	ns	1.7605	0.001
D3ZDT1	Epb41l2	0.002791	1.0328	0.0351	0.7734	ns	0.7182	ns	0.9285	ns
D3ZFB6	Prrt2	0.013560	1.1555	ns	2.6381	<0.0001	1.1707	ns	0.4438	<0.0001
D3ZFQ8	Cyc1	0.001063	0.9540	ns	0.8059	ns	1.3521	ns	1.6778	0.0055
D4A404	Psd3	0.011354	1.1216	ns	0.5643	0.0001	0.9204	ns	1.6310	ns
D4AD15	Eif4g1	0.021211	1.1270	ns	1.2481	ns	0.7258	ns	0.5815	ns
D3ZUX5	Chchd3	0.013245	0.8382	ns	1.1770	ns	0.6042	0.0004	0.5134	<0.0001
D3ZZ21	Ndufb6	0.006064	0.6994	0.0039	0.5843	0.0004	0.8910	ns	1.5248	0.0202
M0RAX4	Gnb5	0.004080	0.9448	ns	0.7620	ns	1.0866	ns	1.4260	0.0621
D4A3V2	Ndufa6	0.000874	0.9306	ns	0.5647	0.0001	0.8406	ns	1.4886	0.03
D4A565	Ndufb5	0.014049	0.8820	ns	0.5956	0.0007	0.8654	ns	1.4530	0.046
D4A6H8	Ctnna2	0.001443	0.9708	ns	1.6786	0.0007	0.8345	ns	0.4972	<0.0001
D4A8U7	Dctn1	0.001303	0.9932	ns	0.6883	0.0228	1.0364	ns	1.5057	0.0253
F1LMH0	Sept_3	0.000981	1.0457	ns	0.6934	0.0265	1.1094	ns	1.6000	0.0081
F1LN88	Aldh2	0.002327	1.0545	ns	0.6360	0.0039	1.1944	ns	1.8779	0.0002
F1LNF1	Hnrnpa2b1	0.019433	1.0574	ns	0.8411	ns	1.0306	ns	1.2254	ns
F1LNK0	Map2	0.002452	1.0305	ns	0.8732	ns	1.0600	ns	1.2140	ns
F1LNY3	Ncam1	0.008813	1.1328	ns	1.9508	<0.0001	0.9379	ns	0.4808	<0.0001
F1LPG3	Pacs1	0.020241	1.0654	ns	0.7806	ns	0.8897	ns	1.1398	ns
F1LPG5	Ndufb4	0.001536	0.8879	ns	0.4128	<0.0001	0.8083	ns	1.9582	<0.0001
F1LPP6	Akap5	0.009399	1.3074	0.0449	1.6623	0.0009	1.0065	ns	0.6055	0.0042
F1LPS8	Pura	0.004160	0.9458	ns	0.5734	0.0002	1.1059	ns	1.9288	<0.0001
F1LQ55	Scp2	0.016330	1.0585	ns	0.5973	0.0008	1.1751	ns	1.9674	<0.0001
F1LQN3	Rtn4	0.009520	0.9131	ns	0.4511	<0.0001	1.2485	ns	2.7676	ns
F1LU71	Auh	0.010860	0.9532	ns	0.7302	ns	0.9428	ns	1.2912	ns
F1LX07	Slc25a12	0.018170	0.8932	ns	0.8085	ns	1.1550	ns	1.4286	ns

F1LXA0	Ndufa12	0.008528	0.8653	ns	0.6908	0.0245	0.9338	ns	1.3518	ns
F1M779	Cltc	0.020911	1.0310	ns	0.9780	ns	1.2814	ns	1.3102	ns
F1M787	Ctnnd2	0.002228	1.0665	ns	1.7555	0.0002	1.0554	ns	0.6012	0.0036
F1M953	Hspa9	0.004235	0.9789	ns	0.8882	ns	1.2608	ns	1.4195	ns
F1M978	Impa1	0.001057	0.9875	ns	0.7036	0.0354	1.1697	ns	1.6624	0.0036
F1MA36	Sptbn2	0.013559	1.0786	ns	0.8810	ns	0.9951	ns	1.1295	ns
F8WFM2	Napb	0.000075	0.9508	ns	0.6577	0.0085	0.8331	ns	1.2668	ns
G3V624	Coro1c	0.011408	1.0095	ns	0.4519	<0.0001	1.1741	ns	2.5979	<0.0001
G3V6D3	Atp5b	0.002654	0.8813	ns	0.8140	ns	1.2193	ns	1.4979	0.0277
G3V6P2	Dlst	0.004135	0.9540	ns	0.7459	ns	1.3251	ns	1.7765	0.0008
G3V741	Slc25a3	0.002374	0.9110	ns	0.7141	0.0468	0.9643	ns	1.3504	ns
G3V7Q6	Psmb5	0.006266	1.0267	ns	0.7271	ns	0.9147	ns	1.2580	ns
G3V8C3	Vim	0.015690	1.6402	<0.0001	0.6388	0.0043	0.7018	0.0215	1.0987	ns
G3V8G4	Bcan	0.004883	1.0331	ns	0.7721	ns	1.2243	ns	1.5856	0.0396
G3V8K2	Gng3	0.008141	0.9180	ns	0.6455	0.0055	0.9468	ns	1.4668	ns
Q5BJ93	Eno1	0.000749	0.9235	ns	0.7813	ns	1.2149	ns	1.5548	0.0141
M0R776	Mrps36	0.000903	1.1193	ns	0.4057	<0.0001	1.2926	ns	3.1864	<0.0001
O08839	Bin1	0.021419	1.0279	ns	0.8102	ns	1.1393	ns	1.4062	ns
O35244	Prdx6	0.001470	0.9548	ns	0.8411	ns	1.2905	ns	1.5343	0.018
O35458	Slc32a1	0.015008	0.8555	0.0047	0.6410	ns	0.5413	<0.0001	0.8445	ns
O35814	Stip1	0.001133	0.9178	ns	0.6655	0.0111	1.1010	ns	1.6545	0.004
O88600	Hspa4	0.010036	1.0653	ns	0.9550	ns	1.3354	ns	1.3984	ns
P00507	Got2	0.001423	0.9667	ns	0.8011	ns	1.0817	ns	1.3503	ns
P02091	Hbb	0.005301	1.4770	0.0014	0.6325	0.0034	1.0129	ns	1.6013	0.0079
P02262	Hist1h2ac	0.002153	0.9835	ns	0.5545	<0.0001	1.0636	ns	1.9181	0.0001
Q45QN0	Gnai2	0.000309	1.0113	ns	0.7146	0.0475	0.9785	ns	1.3692	ns
P05708	Hk1	0.000708	0.9696	ns	0.8166	ns	1.0569	ns	1.2942	ns
P06685	Atp1a1	0.007638	0.8906	ns	0.8044	ns	1.1789	ns	1.4656	0.0401
P06687	Atp1a3	0.019508	0.9722	ns	0.8310	ns	0.9266	ns	1.1151	ns
P07323	Eno2	0.008111	0.9651	ns	0.7742	ns	1.1225	ns	1.4499	0.0478
P07335	Ckb	0.009512	0.9584	ns	0.9032	ns	1.3193	ns	1.4607	0.0424

P07340	Atp1b1	0.000134	0.9463	ns	0.7604	ns	1.1559	ns	1.5201	0.0214
P07483	Fabp3	0.001454	1.0409	ns	0.8441	ns	0.9113	ns	1.0797	ns
Q6LDS4	Sod1	0.000716	1.0027	ns	0.5241	<0.0001	0.9559	ns	1.8239	0.0004
P07825	Syp	0.004418	0.9499	ns	0.5989	0.0008	0.9022	ns	1.5066	0.025
P08082	Cltb	0.014797	0.9565	ns	0.8132	ns	1.0813	ns	1.3297	ns
P08461	Dlat	0.004791	0.9608	ns	0.7826	ns	1.2055	ns	1.5404	0.016
P09117	Aldoc	0.003146	1.0410	ns	0.7497	ns	1.0800	ns	1.4406	ns
P09606	Glul	0.001340	0.9441	ns	0.7609	ns	1.3282	ns	1.7456	0.0012
P09951	Syn1	0.000004	1.1096	ns	0.8288	ns	1.1442	ns	1.3806	ns
P0DP31	Calm2	0.006954	0.9511	ns	0.6661	0.0113	1.2001	ns	1.8016	0.0006
P10111	Ppia	0.002285	0.9124	ns	0.6770	0.0161	1.2099	ns	1.7872	0.0007
P10760	Ahcy	0.017151	1.0798	ns	0.7921	ns	1.1021	ns	1.3913	ns
P10888	Cox4i1	0.016971	0.8091	ns	0.6342	0.0036	0.8646	ns	1.3634	ns
P11348	Qdpr	0.006581	0.8492	ns	0.6417	0.0048	1.1341	ns	1.7674	0.0009
P11507	Atp2a2	0.016255	0.8274	ns	0.6460	0.0056	1.0112	ns	1.5653	0.0124
P11980	Pkm	0.006165	0.9458	ns	0.8773	ns	1.2313	ns	1.4034	ns
P13852	Prnp	0.012896	0.8571	ns	1.6310	0.0015	0.9892	ns	0.6065	0.0044
P15999	Atp5a1	0.004732	0.9142	ns	0.8033	ns	1.1357	ns	1.4138	ns
P16446	Pitpna	0.000001	0.9394	ns	0.4716	<0.0001	1.0798	ns	2.2898	<0.0001
P16617	Pgk1	0.000122	0.9649	ns	0.7162	0.0495	1.2396	ns	1.7308	0.0015
P17105	Itpka	0.000156	1.0761	ns	0.4616	<0.0001	1.0432	ns	2.2597	<0.0001
P18418	Calr	0.003727	1.1172	ns	0.8872	ns	1.1060	ns	1.2467	ns
P18420	Psma1	0.020915	0.9272	ns	1.3031	ns	0.6333	ns	0.4860	ns
P21571	Atp5j	0.002897	0.9559	ns	0.6410	0.0047	0.8198	ns	1.2789	ns
P21575	Dnm1	0.000268	0.9805	ns	0.7918	ns	1.1764	ns	1.4856	0.0319
P21707	Syt1	0.000054	0.9601	ns	0.6711	0.0134	1.1716	ns	1.7456	0.0012
P21913	Sdhb	0.001096	1.1260	ns	0.9116	ns	1.4032	0.0299	1.5392	0.017
P25113	Pgam1	0.003889	0.9816	ns	0.7740	ns	1.2284	ns	1.5871	0.0095
P28480	Tcp1	0.000531	1.0221	ns	0.7356	ns	1.0498	ns	1.4272	ns
P29419	Atp5i	0.000662	0.7130	0.0069	0.4202	<0.0001	0.8323	ns	1.9806	<0.0001
P31044	Pebp1	0.000034	0.9194	ns	0.6625	0.0101	1.3013	ns	1.9641	<0.0001

Q6PCU0	Atp5c1	0.003997	0.9890	ns	0.8167	ns	1.0253	ns	1.2555	ns
P35465	Pak1	0.012900	1.1281	ns	1.0198	ns	1.2658	ns	1.2412	ns
P35565	Canx	0.009790	1.0605	ns	0.8544	ns	1.0730	ns	1.2559	ns
P37377	Snca	0.019524	1.0778	ns	0.7270	ns	0.9383	ns	1.2907	ns
P45592	Cfl1	0.010170	0.9824	ns	0.8025	ns	1.1523	ns	1.4358	ns
P47942	Dpysl2	0.000715	0.8837	ns	0.7249	ns	1.1797	ns	1.6274	0.0057
P47971	Nptx1	0.000074	0.9731	ns	0.4589	<0.0001	1.1198	ns	2.4400	<0.0001
P48500	Tpi1	0.021500	1.0090	ns	0.8331	ns	1.2698	ns	1.5243	ns
P50503	St13	0.004682	1.2174	ns	0.5205	<0.0001	1.0107	ns	1.9418	<0.0001
P54311	Gnb1	0.000712	0.9956	ns	0.7263	ns	1.3174	ns	1.8138	0.0005
P54313	Gnb2	0.005079	1.0242	ns	0.8038	ns	1.2356	ns	1.5372	0.0174
P54921	Napa	0.000124	0.9706	ns	0.5860	0.0004	1.1309	ns	1.9300	<0.0001
P59215	Gnao1	0.000764	0.9230	ns	0.7164	0.0497	0.9313	ns	1.3000	ns
P60841	Ensa	0.000001	0.9388	ns	0.5746	0.0003	0.9270	ns	1.6134	0.0068
P60881	Snap25	0.020714	0.8239	ns	0.6295	ns	0.7426	ns	1.1797	ns
P60905	Dnajc5	0.009092	0.9404	ns	0.6164	0.0018	1.1160	ns	1.8103	0.0005
P62076	Timm13	0.008128	0.8086	ns	0.5927	0.0006	1.1859	ns	2.0008	<0.0001
P62260	Ywhae	0.000084	1.0691	ns	0.8099	ns	1.0358	ns	1.2790	ns
P62762	Vsnl1	0.002715	0.9041	ns	0.7051	0.0368	1.0914	ns	1.5479	0.0153
P62804	Hist1h4b	0.015090	0.9720	ns	0.7072	0.039	1.1756	ns	1.6624	0.0036
Q3ZB97	Ap2b1	0.015430	1.0584	ns	0.9990	ns	1.3315	ns	1.3329	ns
P62963	Pfn1	0.001575	1.0537	ns	0.6897	0.0238	1.2543	ns	1.8185	0.0005
P63012	Rab3a	0.020993	0.9619	ns	0.7976	ns	1.1245	ns	1.4098	ns
P63018	Hspa8	0.000214	0.9579	ns	0.7879	ns	1.1461	ns	1.4546	0.0453
Q9WUW2	Vamp2	0.018888	0.8078	ns	0.6153	0.0017	0.8817	ns	1.4330	ns
P63170	Dynll1	0.006536	0.8158	ns	0.6542	0.0075	1.2587	ns	1.9241	0.0001
P63245	Gnb2l1	0.014406	0.9256	ns	0.4792	<0.0001	0.8999	ns	1.8779	0.0002
P63322	Rala	0.000639	0.8398	ns	0.7443	ns	1.1406	ns	1.5324	0.0185
P63329	Ppp3ca	0.006945	0.9947	ns	0.9489	ns	1.2727	ns	1.3413	ns
P63331	Ppp2ca	0.019454	0.9432	ns	0.8531	ns	1.3090	ns	1.5345	ns
P68035	Actc1	0.012696	0.9255	ns	0.4356	<0.0001	1.0811	ns	2.4818	<0.0001

P68370	Tuba1a	0.008892	0.9420	ns	0.7216	ns	1.1463	ns	1.5886	0.0093
P81155	Vdac2	0.011071	0.8080	ns	0.6788	0.0171	1.0788	ns	1.5892	0.0093
P84076	Hpca	0.011048	0.9327	ns	0.6898	0.0003	1.2299	ns	1.7829	0.0008
P84083	Arf5	0.010884	0.9991	ns	1.7241	ns	1.0168	ns	0.5898	0.0024
P84087	Cplx2	0.000023	0.9972	ns	0.6151	0.0017	1.0529	ns	1.7117	0.0019
P85108	Tubb2a	0.008599	0.9474	ns	0.7705	ns	1.2909	ns	1.6754	0.0031
P85834	Tufm	0.000974	0.8643	ns	0.7169	ns	0.9406	ns	1.3120	ns
P97546	Nptn	0.006064	0.9784	ns	0.6130	0.0015	1.0109	ns	1.6491	0.0043
P97685	Nfasc	0.010265	0.9155	ns	0.8035	ns	1.0489	ns	1.3054	ns
P97846	Cntnap1	0.003288	0.8885	ns	0.4690	<0.0001	1.0365	ns	2.2100	<0.0001
Q05982	Nme1	0.000398	1.0457	ns	0.5775	0.0003	0.8827	ns	1.5286	0.0193
Q06647	Atp5o	0.003517	0.9278	ns	0.6625	0.01	1.2739	ns	1.9229	0.0001
Q3T1K5	Capza2	0.013855	0.9238	ns	0.7819	ns	1.2381	ns	1.5834	0.0099
Q3ZAU6	Rnf14	0.000083	1.0083	ns	2.5251	<0.0001	1.3781	0.0434	0.5458	0.0004
Q45QK6	Gng2	0.000983	1.0342	ns	0.5604	0.0001	1.1184	ns	1.9958	<0.0001
Q52KS1	Pfkm	0.000597	0.9825	ns	0.7466	ns	1.1719	ns	1.5695	0.0118
Q568Z9	Phyhip	0.000621	1.0874	ns	0.8656	ns	1.3178	ns	1.5224	0.0111
Q5BJT9	Ckmt1b	0.000449	0.8880	ns	0.7493	ns	1.1796	ns	1.5743	ns
Q5D023	Dync1li2	0.001327	1.2252	ns	2.2561	<0.0001	1.0273	ns	0.4553	<0.0001
Q5RKI1	Eif4a2	0.000012	1.1019	ns	0.6546	0.0076	1.0421	ns	1.5919	0.0089
Q5XI73	Arhgdia	0.000388	0.9504	ns	0.6344	0.0036	1.1818	ns	1.8629	0.0003
Q5XIT1	Mapre3	0.012880	1.0288	ns	0.7913	ns	0.9802	ns	1.2387	ns
Q62634	Slc17a7	0.002786	1.1110	ns	0.6400	0.0045	0.7886	ns	1.2321	ns
Q62718	Ntm	0.001897	0.9125	ns	0.6214	0.0022	0.8446	ns	1.3591	ns
Q62950	Crmp1	0.013517	0.8970	ns	0.7989	ns	1.2653	ns	1.5837	0.0099
Q63377	Atp1b3	0.000113	0.8798	ns	0.4698	<0.0001	1.2170	ns	2.5905	<0.0001
Q63413	Ddx39b	0.000273	0.9308	ns	0.3730	0.0015	1.1979	ns	3.2116	<0.0001
Q63564	Sv2b	0.020659	0.9594	ns	0.6867	ns	0.8696	ns	1.2664	ns
Q63633	Slc12a5	0.001412	0.9537	ns	0.7574	ns	1.0539	ns	1.3914	ns
Q63716	Prdx1	0.001545	0.9866	ns	0.7228	ns	0.8848	ns	1.2241	ns
Q64548	Rtn1	0.001645	0.9902	ns	0.6129	0.0015	1.0154	ns	1.6566	0.0039

Q66HF1	Ndufs1	0.000018	0.9728	ns	0.7410	ns	1.0720	ns	1.4468	0.0495
Q68FX0	Idh3B	0.000820	0.8844	ns	0.7202	ns	1.1024	ns	1.5307	0.0188
Q68FY0	Uqcrc1	0.002088	1.0478	ns	0.8727	ns	1.1762	ns	1.3478	ns
Q6P3V9	Rpl4	0.000081	0.7940	ns	0.3474	ns	0.7528	ns	2.1669	<0.0001
Q6P7D2	Rnpep	0.002393	1.0640	ns	1.9025	<0.0001	1.0270	ns	0.5398	0.0003
Q6P9V6	Psma5	0.000707	0.9222	ns	0.6498	0.0064	1.0189	ns	1.5679	0.012
Q6P9Y4	Slc25a4	0.000699	0.8995	ns	0.7036	0.0353	0.9902	ns	1.4075	ns
Q6PDU7	Atp5l	0.001504	0.9245	ns	0.6256	0.0026	1.1296	ns	1.8055	0.0006
Q6PDW1	Rps12	0.000074	0.9536	ns	0.4089	<0.0001	0.9337	ns	2.2832	<0.0001
Q6URK4	Hnrnpa3	0.000313	1.1195	ns	0.6609	0.0095	1.0711	ns	1.6206	0.0062
Q6XVN8	Map1lc3a	0.014226	0.7754	ns	0.3039	<0.0001	0.7368	ns	2.4246	<0.0001
Q71RR7	Guk1	0.014633	1.0667	ns	1.2758	ns	0.7495	ns	0.5875	0.0022
Q78P75	Dynll2	0.013955	0.8445	ns	0.6824	0.019	1.1794	ns	1.7284	0.0015
Q7TQ16	Uqcrq	0.008212	0.8424	ns	0.7197	ns	1.0444	ns	1.4511	0.047
Q812E9	Gpm6a	0.011244	1.0183	ns	0.5149	<0.0001	0.9297	ns	1.8056	0.0006
Q8K4Y5	Lgi1	0.001537	0.8990	ns	0.7474	ns	1.0378	ns	1.3886	ns
Q9EPH8	Pabpc1	0.014480	0.8802	ns	0.5900	0.0005	1.1328	ns	1.9200	0.0001
Q9EQS0	Taldo1	0.001774	0.9553	ns	1.9420	<0.0001	1.1817	ns	0.6085	0.0047
Q9ER34	Aco2	0.000204	1.0112	ns	0.7878	ns	1.1666	ns	1.4808	0.0338
Q9ERH3	Wdr7	0.003949	0.7866	ns	0.3605	<0.0001	0.8920	ns	2.4742	<0.0001
Q9JHL4	Dbnl	0.010594	1.3598	0.0166	1.5724	0.0039	1.0080	ns	0.6411	0.0133
Q9JHY2	Sfxn3	0.011218	0.7944	ns	0.7911	ns	1.0655	ns	1.3469	ns
Q9JJ19	Slc9a3r1	0.000482	1.2331	ns	2.1402	<0.0001	1.2915	ns	0.6035	0.0039
Q9R063	Prdx5	0.000695	0.9039	ns	0.7360	ns	1.2734	ns	1.7301	0.0015
Q9R0I8	Pip4k2a	0.003146	1.1120	ns	0.5159	<0.0001	0.9869	ns	1.9128	0.0001
Q9WV97	Timm9	0.001724	1.1003	ns	0.4385	<0.0001	0.8242	ns	1.8799	0.0002
Q9Z0W5	Pacsin1	0.010111	1.1281	ns	0.9032	ns	1.1556	ns	1.2794	ns
Q9Z2L0	Vdac1	0.006548	0.8253	ns	0.7294	ns	1.0859	ns	1.4887	0.038
