# Supplementary figures and images for: Global Proteome Profiling of the Temporal Cortex of Female Rats Exposed to Chronic Stress and the Western Diet
Source: Nutrients. 2022 May 5;14(9):1934. doi: 10.3390/nu14091934 (PMC9103025; doi:10.3390/nu14091934)

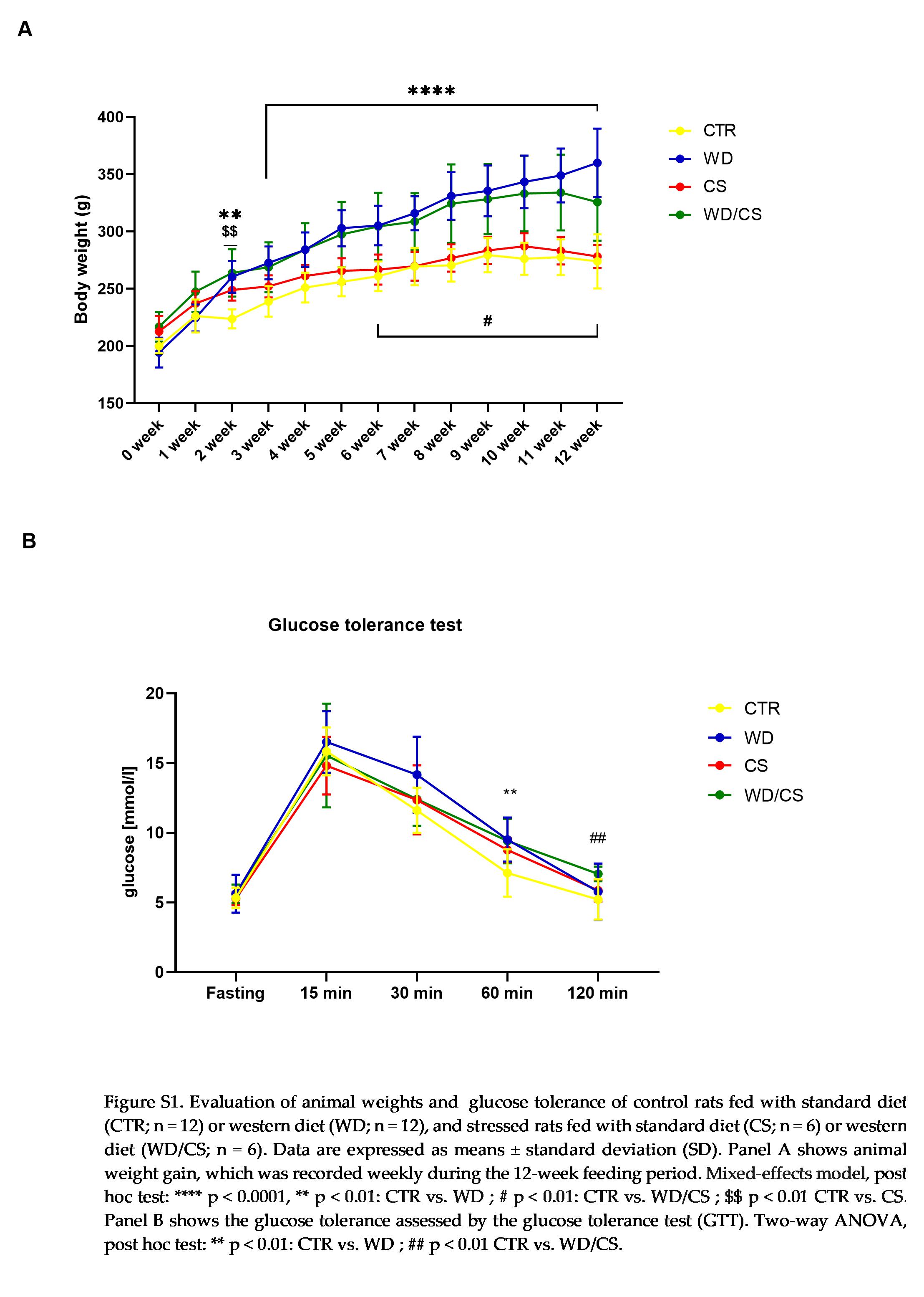

Supplement: Supplementary file 1 [file nutrients-14-01934-s001.zip › Figure S 1.jpg]
